# Association between Sense of Belonging and Loneliness among the Migrant Elderly Following Children in Jinan, Shandong Province, China: The Moderating Effect of Migration Pattern

**DOI:** 10.3390/ijerph19074396

**Published:** 2022-04-06

**Authors:** Guangwen Liu, Shixue Li, Fanlei Kong

**Affiliations:** 1 Centre for Health Management and Policy Research, School of Public Health, Cheeloo College of Medicine, Shandong University, Jinan 250012, China; liugw16@mail.sdu.edu.cn; 2 NHC Key Lab of Health Economics and Policy Research, Shandong University, Jinan 250012, China

**Keywords:** migrant elderly following children, sense of belonging, loneliness, migration pattern, inter-provincial migration

## Abstract

Background: Driven by accelerating population aging and migration, the number of older migrants has increased rapidly in China. Those who moved to cities to look after grandchildren were referred to as the migrant elderly following children (MEFC). This study aims to examine the relationship between sense of belonging and loneliness and explore the moderating effect of migration pattern among the MEFC in China. Methods: The study included 656 MEFC aged 60 years and above. Loneliness was evaluated by the eight-item University of California Los Angeles Loneliness Scale (ULS-8). Sense of belonging and migration pattern were measured using a self-designed questionnaire. Hierarchical multiple regression was conducted to test the proposed association and moderating effect. A margins plot was introduced to illustrate this effect. Results: The average ULS-8 score was 12.82 ± 4.05, revealing a low level of loneliness. A weak sense of belonging was related with a higher level of loneliness (β = 0.096, *p* = 0.014). Migration pattern was found to exacerbate this association (β = 0.138, *p* = 0.026), especially for the elderly who migrated across provinces. Conclusions: Sense of belonging was correlated with loneliness, and the moderating role of migration pattern was established. Both policymakers and the adult children of inter-provincial migrant elderly should focus on this special subgroup.

## 1. Introduction

Population aging, the phenomenon in which an increasing proportion of people progress into their later lives, has become a worldwide trend. As the largest developing country, China has also witnessed a sharp increase in the aging population. Statistics from the latest National Population Census indicated that there are 264 million elderly people aged 60 and above in mainland China, accounting for 18.70% of the entire population [1]. Although the decision on the three-child policy (a Chinese family planning policy which allows a couple to have three kids) and relevant supporting measures had been made in May 2021 to promote long-term and balanced population development [2], the rapid population aging trend cannot be reversed in the short term. Moreover, as the second largest economy all over the world, China has undergone accelerated urbanization and industrialization, which has brought about the largest migrant population across the globe. In 2020, there were 375 million migrants, a 70% increase over the past decade. Among them, 124 million were inter-provincial and 251 million were intra-provincial [3]. Driven by the rapid development in aging and migration, the scale of migrant elderly has trended similarly, reaching 13.04 million in 2015, and accounting for 5.9% of all migrants [4]. A previous study revealed that unlike the migrant elderly in Western countries, almost 80% of Chinese migrant elderly moved to cities to provide care for grandchildren and retire closer to their adult children [5]. These elderly migrants have been defined as the migrant elderly following children (MEFC) in the current study.

Older migrants are one of the most vulnerable populations. The group has attracted considerable attention from researchers at home and abroad. Studies outside of China on international older immigrants have focused primarily on the migration-related adverse mental health caused by distinct differences in language and cultural environment [6,7,8]. Differing inclinations between Western countries and China have added significance to studies on the MEFC, especially studies that explored the mental health problems among them.

Loneliness is defined as “an aversive emotional response to a perceived discrepancy between one’s desired and actual social relationships” [9]. Older adults have been shown to be at high risk of loneliness and thus should be considered a crucial subgroup in many research fields [10]. Apart from other sociodemographic variables and health-related attributes in common with their local counterparts [11], migrant elderly have reported independent risk factors, the foremost being the social factors, such as the separation of old friends or peers and lost contact with their existing social networks [12]. As is mentioned above, limited attention was paid on the loneliness among older migrants inside of China as there was similar linguistic and cultural background to a large degree within the same country. Notably, as migration distances increased, they may have had to change their lifestyles and adapt to new living environments [13], which at least influenced residential satisfaction and a sense of home [14]. Loneliness among the MEFC in China remained understudied.

A sense of belonging refers to “the experience of personal involvement in a system or environment so that persons feel themselves to be an integral part of that system or environment” [15]. Former scholars have theoretically explored the association between sense of belonging and loneliness and demonstrated that the lack of a sense of belonging provided by wider social circles was the underlying reason for social loneliness [16]. This relationship has been supported by empirical studies as well [17]. An investigation among 357 children of Chinese migrant workers found a negative correlation between the sense of belonging and loneliness, meaning that children who did not think they belonged to their school or city felt lonelier [18]. A weak sense of belonging was also found to be associated with loneliness among those aged 50–80 years in the Western Finland study [19]. However, studies concentrating on the relationship between a sense of belonging and loneliness among the older migrants were scarce. Most articles pertaining to elderly migrants have been focused on international expats, such as the Turkish and Moroccan elderly in the Netherlands [20]. To the best of our knowledge, no research has explored the effect of a sense of belonging on loneliness among Chinese elderly migrating internally.

Migration patterns basically involved two aspects: spatial pattern (e.g., intra-provincial or inter-provincial migration) and membership pattern (e.g., migrating alone, with spouse, or with children). Extant studies have examined the link between migration pattern and loneliness or other adverse mental health outcomes [21,22,23]. To be specific, Zhong et al. explored the membership pattern of migration and concluded that migrant workers who migrated alone were 1.97 times more likely to suffer from loneliness than those who migrated with all family members [24]. However, in terms of the spatial pattern, more studies emphasized that international older migrants from different countries of origin exhibited varying prevalence of loneliness [25]. There has been no evidence showing that the spatial pattern of migration was related to loneliness among Chinese internal older migrants.

In addition, different spatial patterns represented different migration distance. Long-distance migration generates many problems, including complicated medical insurance policies, discomfort from climate change, and language barriers due to dialects [26], all of which should be solved at the earliest. The lack of a sense of belonging was one of the most important challenges because it was related to life satisfaction [27], subjective wellbeing [28], and quality of life [29] among the MEFC. Therefore, the spatial pattern of migration was the focus of the current study, and it was that the migration pattern moderated the relationship between a sense of belonging and loneliness.

In summary, although several studies have examined the association between the sense of belonging and loneliness among international elderly migrants, none have testified this relationship among the MEFC in China. Moreover, no researcher has explored the effect of migration pattern, especially spatial pattern, on the sense of belonging-loneliness link. Thus, this study aims to uncover the prevalence of loneliness among the MEFC in Jinan, Shandong Province, examine the association between the sense of belonging and loneliness, and verify whether there is a moderating effect exerted by the spatial pattern on this association. We hypothesized that there was a negative relationship between the sense of belonging and loneliness, and the spatial pattern of migration would serve as a significant moderator on this relationship.

## 2. Materials and Methods

### 2.1. Data Collection and the Research Subjects

The data was collected in Jinan City, Shandong Province, China in August 2020. The gross domestic product of Shandong Province was 9.3 trillion Chinese Yuan (≈1.3 trillion US$) in 2021 [30]. The total population of Shandong Province was 101 million by the end of 2020 according to the Seventh National Census [31]. Jinan City is the capital of Shandong Province, one of the Chinese eastern provinces. The gross domestic product of Jinan in 2020 was 1.01 trillion Chinese Yuan (≈157,285.51 million US$) [32]. Jinan governed 10 districts and 2 counties (132 sub-districts and 29 towns) until July 2020 [33]. By the end of 2019, Jinan had a total of 8.91 million local residents, an increase of 0.78% compared to the end of 2018, while the number of registered population was 7.98 million, an increase of 1.46% [34]. Jinan had 2.9 million migrants in 2019 [35], and the moving population who are 60 years old or above and follow their children to Jinan City are included in this study. To choose the study subjects, multi-stage cluster random sampling was conducted. In stage 1, three of ten districts were selected as the primary sampling units (PSUs), considering the economic development and the geographic location. In stage 2, a total of three sub-districts were chosen as the secondary sampling units (SSUs) from each PSU; that is, one sub-district was selected from each of the districts chosen previously. In stage 3, three communities were chosen from the SSUs; that is, one community was selected from each of the sub-districts chosen previously. All the migrant elderly who lived in the above communities, were 60 years or above, and followed their children to Jinan City constituted the total study sample.

Thirty-two college students were recruited as investigators after the training about the background of the whole study, questionnaire content, and the technique on social survey. Twenty-minute face-to-face data collection processes were conducted between the investigators and subjects. Some of the interviews were held in participants’ home after their permission while others were held in public areas of the communities. Before every interview, the consent to participate were obtained by asking the respondents whether they had time and were willing to join the survey after the introduction of the background and the purpose of the research.

At first, a total of 670 migrant elderly who followed their children were selected and interviewed. However, 14 of them were removed from the sample because of obvious logical errors or uncompleted questionnaires. Finally, 656 older adults were included in the database.

### 2.2. Measurements

#### 2.2.1. Dependent Variable

Loneliness was measured by the short version of the University of California Los Angeles Loneliness Scale (ULS-8). Russell created the initial University of California Los Angeles (UCLA) Loneliness Scale with 20 items in 1978 [36] and revised the UCLA Loneliness Scale to counter the possible effects of response bias in 1980 [9]. Hays and DiMatteo further selected eight items from the revised UCLA Loneliness Scale using exploratory factor analysis and designed the ULS-8 [37]. Some scholars have translated the ULS-8 into Chinese and verified the reliability and validity of the scale [38,39]. Each item was rated on a 4-point Likert scale ranging from 1 (“never”) to 4 (“always”). The scale also encompassed two reverse-coded items. Thus, the total ULS-8 score ranges from 8 to 32; the higher the score, the lonelier the elderly are.

#### 2.2.2. Independent Variables

##### Sense of Belonging

Sense of belonging was evaluated by the statement “I consider myself a local citizen.”, an item used in the China Migrants Dynamic Survey (CMDS) [40]. Participants were asked to select one response on a 4-point Likert scale that ranged from 1 (“totally disagree”) to 4 (“totally agree”). As Nyqvist et al. did in a previous study [19], those who chose “totally disagree” and “disagree” were deemed to have a weak sense of belonging, while those who answered “basically agree” and “totally agree” were deemed to have a strong sense of belonging.

##### Migration Pattern

Three spatial patterns of migration among the MEFC were included in the current research. Participants who moved from other districts or counties in Jinan City were categorized by “across districts or counties”. Those who migrated from other prefecture-level cities in Shandong Province were divided into “across prefecture-level cities’’. Migrant elderly who moved to Jinan from other provinces were classified into “across provinces”.

##### Covariables

Basic sociodemographic characteristics such as gender, age, marital status, educational level, monthly income, and health status of the respondents were included as covariables. Gender was coded as male or female; age was coded as 60 to 69 years old, 70 to 75 years old, or over 75 years old; marital status was coded as currently married or single (including unmarried, divorced, and widowed); educational level was coded as illiterate, primary school, or middle school or above; and monthly income was classified into four groups by quartile. As for health status, participants were asked whether they suffered from hearing impairment, had developed any chronic diseases, and received outpatient service or inpatient service in the last year. Responses to these questions were “yes” or “no”. Considering living arrangement, participants were asked if they shared a bedroom with others. Response to this question was “live alone” or “live with others” (such as the spouse, children, grandchildren, etc.). With a focus on the MEFC, the study also controlled for time since migration, willingness to migrate, Hukou, and temporary residential permit. Time since migration was coded as under five years and five years or above; willingness to migrate was first evaluated using a 5-point Likert scale ranging from 1 (“totally unwilling”) to 5 (“totally willing”) and then merged into “unwilling” (including totally unwilling, partially unwilling, and neutral) and “willing” (including partially willing and totally willing); Hukou was coded as rural or urban; and temporary residential permit was coded as yes or no.

### 2.3. Statistical Analysis

Mean and standard deviation (for the continuous variables) and percentage (for categorical variables) were used to describe the sociodemographic characteristics of the sample. *t*-test and one-way ANOVA were conducted to analyze variations in ULS-8 scores among the different groups. Hierarchical multiple regression analysis was used to explore the association between the sense of belonging and loneliness as well as the moderating effect of migration pattern. Sense of belonging was the only variable introduced in Model 1. Confounders were further introduced in Model 2 to test whether there was a relationship between the sense of belonging and loneliness. To examine the moderating role of migration pattern in the relationship, the interaction term (sense of belonging × migration pattern) was then included in Model 3. Furthermore, a margins plot was employed to illustrate the prediction of loneliness based on the sense of belonging categories and migration pattern. Statistical differences were considered significant when *p* ≤ 0.05. All analyses were performed using SPSS 22.0 (IBM Corp., Armonk, NY, USA).

### 2.4. Ethical Considerations

Medical ethics approval of this study was approved by the Ethical Committee of School of Public Health, Shandong University (No. 20180225).

## 3. Results

### 3.1. Basic Information of The Participants

The basic characteristics of the study sample are presented in Table 1. A total of 656 elderly individuals were included in the study, with a mean age of 66.19 ± 4.53 years old. They got an average score of 12.82 ± 4.05 when answering the ULS-8. To be specific, the average score among the 60–69, 70–75, and over 75 age group were 12.82 ± 4.03, 12.18 ± 3.92, and 14.42 ± 4.40, respectively. A weak sense of belonging was noted in 39.2% of respondents. When it comes to the spatial pattern of migration, 67.2% of participants moved across prefectural-level cities; migration across districts or counties was less frequent (146 respondents, 22.3%). Approximately 10% of participants migrated across provinces. For the sociodemographic features, most of them were female (63.7%), currently married (88.9%), received middle school education or above (48.2%), were willing to migrate to Jinan City (90.9%), had a rural Hukou (87.5%), did not get a temporary residential permit (64.8%), did not suffer from hearing impairment (88.6%), developed no chronic disease (55.6%), and did not receive outpatient service (74.8%) and inpatient service (84.6%) over the last year. A total of 71.3% of individuals shared a bedroom with others. Half of the study participants had moved to Jinan city five years ago or above. Furthermore, univariate analysis was conducted to compare variations in loneliness. Significant differences in loneliness were found between age, monthly income, time since migration, willingness to migrate, temporary residential permit, hearing impairment, chronic diseases, outpatient service last year, and sense of belonging among the MEFC in Jinan, Shandong Province, China.

### 3.2. Association between Sense of Belonging and Loneliness

Table 2 shows that the MEFC with a weak sense of belonging reported a higher level of loneliness than those with a strong sense of belonging (β = 0.154, *p* < 0.001), and the average increase in the score of ULS-8 was 0.154 (Model 1). Model 2 further included covariables and found that sense of belonging was still significantly associated with loneliness among the MEFC in Jinan, Shandong Province, China (β = 0.096, *p* = 0.014).

### 3.3. The Moderating Effect of Migration Pattern on the Association between Sense of Belonging and Loneliness

**Figure 1:**
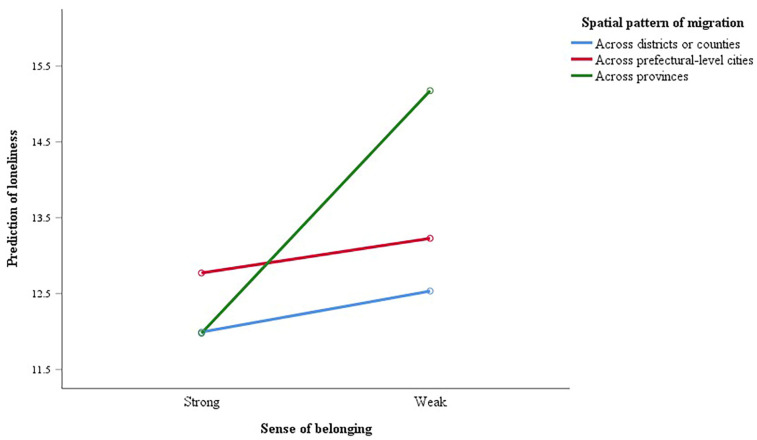
Interaction between sense of belonging and spatial pattern of migration in the prediction of loneliness (adjusted for the covariables in hierarchical multiple regression analysis).

As shown in Table 2, the migration pattern of the subjects was also included in Model 2, revealing no significant relationship between migration pattern and loneliness. Model 3 introduced the interaction term (sense of belonging × migration pattern) to examine whether there was a moderating role of migration pattern played in the correlation between sense of belonging and loneliness. It turned out that migration pattern increased the probability of the older individuals with a weak sense of belonging exacerbating their loneliness (β = 0.138, *p* = 0.026). Figure 1 provides clear evidence that migrating to Jinan from other provinces moderated the association between sense of belonging and loneliness among the MEFC in Jinan, Shandong Province, China.

## 4. Discussion

This study offers new empirical evidence concerning the association between the sense of belonging and loneliness, as well as about the moderating role that spatial pattern of migration played in this association among the MEFC in Jinan, Shandong Province, China. It was found that a weak sense of belonging was significantly related to higher perceived loneliness. Meanwhile, the moderating effect existed only when the spatial type was across provinces.

### 4.1. The Level of Loneliness among the MEFC in Jinan, Shandong Province

The mean score of ULS-8 among Chinese MEFC populations was 12.82 ± 4.05, indicating a slightly lower level of loneliness than that reported in a study of Italian community-dwelling older individuals (13.10 ± 6.90) [41] and a much lower level in comparison with that reported in Singaporean community-dwelling elderly (14.43 ± 4.97) [42]. The potential explanation for this difference may lie in that the ability to migrate depends on lower disability levels and fewer limitations on daily activities [43], factors that further contribute to relieving geriatric loneliness among the study participants. In addition, the MEFC live together with their adult children, making them more likely to experience the happiness of a family union and thus mitigating their loneliness. However, this study’s average score was relatively higher compared with another research among rural elderly in Shandong Province (11.59 ± 4.59) [44]. One possible explanation for this divergence was the different socioeconomic status between these sample cities.

### 4.2. Relationship between Sense of Belonging and Loneliness

Sense of belonging was negatively associated with loneliness in the current study, which was consistent with several other studies. Prieto-Flores et al. found that sense of belonging exerted a negative effect on loneliness among the elderly living in communities, and the result was robust for those living in residential care facilities [45]. De Jong Gierveld et al. explored the effect of ethnic-cultural background on loneliness among Canadian older immigrants at the micro, meso, and macro levels. They found that the sense of belonging to the local community was the only significant indicator relevant to loneliness among the meso-level variables after adjusting for variables at other levels and for sociodemographic characteristics [46]. Moreover, one study in Luxembourg revealed that the weak cultural belonging resulted in low intergenerational belonging; both weak cultural belonging and low intergenerational belonging were strong risk factors for perceived loneliness among Portuguese immigrants [47].

Acculturation strategies determined how a weak sense of belonging functioned as a risk factor for loneliness. Older international immigrants who neither retained their heritage culture from their countries of origin nor adapted to the new culture in host countries would adopt marginalization strategies [48], and thus yielded lower levels of sense of belonging and loneliness [20]. For the MEFC in China, considering the prevailing Confucian culture within this country, what they are confronted with is which acculturation strategy to adopt among various regional cultures, especially in different provinces. Hence, the spatial migration pattern of the MEFC could serve as critical moderators.

### 4.3. The Moderating Role of Migration Pattern in the Relationship between Sense of Belonging and Loneliness

More than two thirds of the MEFC followed adult children to Jinan City from other prefecture-level cities because Jinan is the capital city of Shandong Province and had the second largest GDP in Shandong Province in 2020 [49], which attracted many migrants to move here for better-paid jobs, personal development, or taking care of the grandchildren from other cities. Thus, the migration pattern of across prefecture-level cities was the main pattern of the MEFC. This study also found that the spatial pattern of migration moderated the relationship between the sense of belonging and loneliness, rather than being directly related to the MEFC’s loneliness. Specifically speaking, the moderating effect of inter-provincial pattern was significant, whereas the across prefectural-level cities and districts or counties were not significant. Extant research has demonstrated that a shorter migration distance (measured by migration across counties) could help elevate subjective well-being instead of loneliness among moving population [50]. In our current study, these findings could be interpreted as follows. First, most elderly Chinese people migrating inter-provincially left inland or frontier provinces for metropolitan areas in coastal provinces [51]. They may have endured drastic changes in lifestyles, customs, dialects, and basic amenities, resulting in notable psychological gaps and low levels of belongingness to host provinces. Second, the majority of the migrant elderly who moved across provinces were likely to dwell in the new destinations temporarily or circularly [52], especially the MEFC who often treated assisting adult offspring in taking care of grandchildren as a responsibility or task. Once the grandchildren are grown up, they might intend to return to their original locations. This situation prevailed for long-distance migrants. Third, policies on medical insurance were similar within one province in general. When older migrants tried to seek medical services for illness trans-provincially, a series of barriers including complex expenditure settlement procedures, low reimbursement rates, and few medical insurance designated hospitals would appear [53]. Previous studies have found that geographical disparity in different kinds of medical insurance (e.g., Basic Medical Insurance System for Urban and Rural Residents or Basic Medical Insurance System for Urban Employees) between place of the insurance and place of residence made the migrant elderly experience the inequity of health services utilization [54]. Thus, health inequity also prevented the MEFC from recognizing themselves as local citizens. A weak sense of belonging, based on all these circumstances, was found to be more detrimental to loneliness among the MEFC coming from other provinces.

### 4.4. Association between Other Significant Variables and Loneliness

Age was found to be associated with loneliness among the MEFC. Specifically speaking, those who aged 70–75 years old reported a lower score on ULS-8 compared to those aged 60–69 years. Findings in the current study reflected that the age distribution of loneliness was U-shaped, meaning that the level of loneliness declined since the middle age and the tendency of decline would not stop until the oldest old age (>80 years) [55,56]. It was found that the elderly at the second quartile of income level was lonelier than those at the first quartile. One probable reason might be that older migrants were more likely to depend on offspring economically than the local elderly [57]. Therefore, the monthly income may not represent the actual situation. Participants who migrated to Jinan City for five years or above perceived a lower level of loneliness because they have been familiar with the lifestyles and living environment. Older individuals who did not get a temporary residential permit were lonelier because it was inconvenient for them to get equal access to many public services such as healthcare without it compared to their local counterparts [58]. Finally, hearing impairment was found to deteriorate perceived loneliness, which was in line with former studies [59].

### 4.5. Implications

Firstly, policymakers should enact measures to reduce the difficulty related to reimbursement faced by the MEFC whose medical insurance was not obtained originally in the province to which they have migrated and provide inter-provincial MEFC with equal access to social services compared with their intra-provincial counterparts. These actions would alleviate the adverse effect of a weak sense of belonging on loneliness among the MEFC participants moving across provinces. Secondly, findings of this study could also attract more Chinese scholars to concentrate on the level of loneliness among older migrants, especially the sociocultural contributors, and make it possible to conduct comparative research between subgroups of older migrants in the future.

### 4.6. Limitations

This study has several limitations. Firstly, the cross-sectional design could only test the correlation between the sense of belonging and loneliness but was not able to prove a causal relationship. Secondly, although closely related to the sense of belonging, one question could not measure this variable comprehensively. A scale concerning sense of belonging should be used in future study. Thirdly, many researchers have studied the loneliness in older adults, thus more efforts should be made to understand the loneliness in middle-aged adults and the age differences of loneliness in the future, as well as the loneliness of the left-behind elderly. Fourthly, the data were self-reported, which made recall and reporting bias inevitable. Fifthly, differences in the household registration system and medical insurance policies between cities and provinces in China were much more obvious than those in any other country, which limited the extrapolation of study results.

## 5. Conclusions

The level of loneliness among the MEFC in Jinan, Shandong Province, China was found to be relatively low. The current study clarified the empirical association between the sense of belonging and loneliness and found that the spatial pattern of migration moderated this relationship. Inter-provincial older migrants ought to be recognized as a key population for social and health policies.

## Figures and Tables

**Table 1:** Description and univariate analysis of loneliness among the MEFC in Jinan, Shandong Province, China.

Variables	N (%)	Mean Score of ULS-8 (SD)	t/F	*p*-Value
Total	656 (100)	12.82 (4.05)		
Gender			0.608	0.436
Male	238 (36.3)	12.66 (4.14)		
Female	418 (63.7)	12.92 (4.00)		
Age			3.354	0.036
60–69	552 (84.1)	12.82 (4.03)		
70–75	73 (11.1)	12.18 (3.92)		
Over 75	31 (4.7)	14.42 (4.40)		
Marital status			1.592	0.112
Currently married	583 (88.9)	12.73 (4.03)		
Single ^a^	73 (11.1)	13.53 (4.16)		
Educational level			2.643	0.072
Illiterate	196 (29.9)	13.36 (4.18)		
Primary school	144 (22.0)	12.75 (3.94)		
Middle school or above	316 (48.2)	12.52 (4.00)		
Monthly income ^b^			7.861	<0.001
Q1	199 (30.3)	12.90 (4.20)		
Q2	158 (24.1)	13.94 (4.45)		
Q3	138 (21.0)	12.65 (3.51)		
Q4	161 (24.5)	11.78 (3.62)		
Time since migration			4.175	<0.001
Under five years	328 (50.0)	13.48 (4.07)		
Five years or above	328 (50.0)	12.17 (3.93)		
Willingness to migrate			2.166	0.031
Unwilling	60 (9.1)	13.90 (4.18)		
Willing	596 (90.9)	12.71 (4.03)		
Hukou			0.364	0.716
Rural	574 (87.5)	12.84 (4.10)		
Urban	82 (12.5)	12.67 (3.74)		
Temporary Residential Permit			4.107	<0.001
Yes	231 (35.2)	11.95 (3.79)		
No	425 (64.8)	13.30 (4.12)		
Living arrangement			2.142	0.033
Living alone	188 (28.7)	13.36 (4.52)		
Living with others	468 (71.3)	12.61 (3.83)		
Hearing impairment			2.470	0.014
No	581 (88.6)	12.68 (3.92)		
Yes	75 (11.4)	13.91 (4.86)		
Chronic diseases			2.011	0.045
No	365 (55.6)	12.54 (3.85)		
Yes	291 (44.4)	13.18 (4.27)		
Outpatient service last year			2.299	0.022
No	491 (74.8)	12.61 (3.96)		
Yes	165 (25.2)	13.45 (4.26)		
Inpatient service last year			1.439	0.151
No	555 (84.6)	12.73 (4.04)		
Yes	101 (15.4)	13.36 (4.08)		
Migration pattern			1.426	0.234
Across districts/counties	146 (22.3)	12.32 (3.66)		
Across prefecture-level cities	441 (67.2)	12.92 (4.16)		
Across provinces	66 (10.1)	13.36 (4.04)		
Sense of belonging			3.981	<0.001
Strong	399 (60.8)	12.65 (3.92)		
Weak	257 (39.2)	15.44 (5.03)		

Note: SD: Standard deviation ^a^: Single included those who were unmarried (10, 1.5%), divorced (5, 0.8%), and widowed (58, 8.8%); ^b^: Q1 was the poorest and Q4 was the richest.

**Table 2:** Association between sense of belonging and loneliness among the MEFC in Jinan, Shandong Province, China.

Variables	Model 1		Model 2		Model 3	
	β	*p*-Value	Β	*p*-Value	β	*p*-Value
Main terms						
Sense of belonging						
Strong	Ref.		Ref.		Ref.	
Weak	0.154	<0.001	0.096	0.014	0.062	0.491
Migration pattern						
Across districts/counties			Ref.		Ref.	
Across prefectural-level cities			0.080	0.067	0.090	0.080
Across provinces			0.084	0.053	0.001	0.992
Interaction term						
Sense of belonging × migration pattern						
Sense of belonging × across districts/counties					Ref.	
Sense of belonging × across prefectural-level cities					−0.006	0.951
Sense of belonging × across provinces					0.138	0.026
Covariables						
Age						
60–69			Ref.		Ref.	
70–75			−0.077	0.048	−0.081	0.037
Over 75			0.047	0.233	0.043	0.274
Monthly income ^a^						
Q1			Ref.		Ref.	
Q2			0.126	0.004	0.134	0.002
Q3			−0.015	0.724	−0.011	0.792
Q4			−0.060	0.174	−0.059	0.183
Time since migration						
Under five years			Ref.		Ref.	
Five years or above			−0.110	0.004	−0.117	0.002
Willingness to migrate						
Unwilling			Ref.		Ref.	
Willing			−0.064	0.086	−0.068	0.068
Temporary Residential Permit						
Yes			Ref.		Ref.	
No			0.130	0.001	0.135	0.001
Living arrangement						
Living alone			Ref.		Ref.	
Living with others			−0.069	0.066	−0.072	0.054
Hearing impairment						
No			Ref.		Ref.	
Yes			0.095	0.017	0.096	0.016
Chronic diseases						
No			Ref.		Ref.	
Yes			0.036	0.344	0.034	0.373
Outpatient service last year						
No			Ref.		Ref.	
Yes			0.069	0.073	0.074	0.053
F	15.849	<0.001	6.127	<0.001	5.889	<0.001
$R_{c}^{2}$	0.022		0.105		0.113	
$\Delta R_{c}^{2}$	-		0.083		0.008	

Note: β: Standardized coefficients. ^a^: Q1 was the poorest and Q4 was the richest.

## Data Availability

The datasets used and analysed in this study are available from the corresponding author on reasonable request.
